# Novel pyroptosis-immune-related lncRNA signature exhibits a distinct immune cell infiltration landscape in breast cancer

**DOI:** 10.3389/fimmu.2024.1522327

**Published:** 2025-01-21

**Authors:** Dedi Kong, Hongju Cheng, Meihong Wang

**Affiliations:** ^1^ Shandong Qianfoshan Hospital, Cheeloo College of Medicine, Shandong University, Jinan, Shandong, China; ^2^ Breast and Thyroid Surgery, Jining No 1 People’s Hospital, Jining, Shandong, China; ^3^ Physiology Teaching and Research Office, Jining Medical College, Jining, Shandong, China; ^4^ Hematology Department, Jining No 1 People’s Hospital, Jining, Shandong, China

**Keywords:** pyroptosis, immune infiltration, lncRNA, breast cancer, prognosis

## Abstract

**Introduction:**

This study investigated pyroptosis- and immunity-related long non-coding RNAs (lncRNAs) to identify promising therapeutic targets for breast cancer (BC), and constructed lncRNA signatures to determine the prognosis and immunotherapy responses of BC patients.

**Methods:**

Pearson’s correlation coefficient was used to identify pyroptosis- and immune-related differentially expressed lncRNAs (DE-pyrolncRNAs and DE-ImmlncRNAs, respectively). The Cancer Genome Atlas dataset was allocated to training and testing subsets. Prognostic lncRNA signatures were derived based on the training subset using univariate Cox regression analysis and Least Absolute Shrinkage and Selection Operator methods. Stepwise Cox regression was used to refine these signatures and to select the optimal lncRNA signature. The median risk score of the training subset was applied as a threshold to divide patients into high-risk (HR) and low-risk (LR) groups. The Wilcoxon test was used to reveal differences in immune scores, cell types, functions, and checkpoint genes between these groups. Single-cell sequencing data from GSE176078 were used to validate the immune cell infiltration landscape of the identified lncRNA signatures.

**Results:**

We identified a six-lncRNA pyroptosis-immune signature comprising MAPT.AS1, CTA.384, D8.34, RP11.561, I11.3, HID1.AS1, AC097713.3, and USP2.AS1. Patients in the HR group demonstrated inferior prognoses in the training, testing, and full datasets (P=3.622e-07, P=3.736e-03, and P=1.151e-08, respectively). Immune scores were significantly enhanced in the LR group, whereas tumor purity was elevated in the HR group. Fifty-eight immune scores showed significant differences between the groups (P<0.05). Immune function (APC coinhibition, CCR, and checkpoints) more significantly impaired in the HR group. Expression levels of 38 immune checkpoint genes, including KIR2DS4, KIR3DL2, CD40LG, KIR3DL1, and PDCD1, were significantly higher in the LR group. Conversely, the TDO2, PVR, and CD276 levels were elevated in the HR group. Single-cell sequencing data from GSE176078 showed sparse T cell, B cell, myeloid, and plasmablast clusters in the HR group, whereas the LR group displayed significant clustering of B cells, myeloids, and plasmablasts.

**Conclusion:**

The six-lncRNA pyroptosis-immune signature effectively predicted BC prognosis and highlighted distinct immune cell infiltration patterns. This holds promise for evaluating immunotherapy responses and guiding therapeutic target identification in BC.

## 1 Introduction

In 2020, breast cancer (BC) became the most prevalent form of cancer and the leading cause of cancer-related deaths among women (1). Given BC’s highly heterogeneous nature of BC (2), current treatment strategies are inadequate to address key issues, such as tumor metastasis, recurrence, and drug resistance (3). This underscores the urgent need to identify crucial genes involved in BC, explore the underlying molecular mechanisms, and identify novel and effective treatment options.

Pyroptosis, a form of programmed cell death, triggers inflammation by releasing signaling molecules and cytokines, resulting in robust inflammatory responses and immune activation (4, 5). Unlike apoptosis, pyroptosis causes cell swelling, plasma membrane rupture, chromatin fragmentation, and release of proinflammatory substances (6). Recent research indicates that pyroptosis is crucial for tumor proliferation, invasion, and metastasis and is regulated by various molecules. It has been linked to the progression and treatment of multiple cancers (7–9), including breast (10), colon (11), ovarian (12), lung (13), gastric (14), and hepatocellular carcinoma (15).

Long noncoding RNAs (lncRNAs, >200 nucleotides in length) participate in cell proliferation, apoptosis, and migration (16–18). Several lncRNAs have high tissue- and cell-type specificity and regulate the malignant function of BC cells and multidrug resistance, making them potential therapeutic targets (16, 18). Recent evidence suggests that lncRNAs involved in pyroptosis are associated with other cancers (19, 20). For example, in triple-negative BC, DDP-induced pyroptosis involves the MEG3/NLRP3/caspase-1/GSDMD pathway (21), and the MALAT1/miR-124/SIRT1 axis has been implicated in pyroptosis, offering therapeutic targets (22).

Accumulating evidence has shown that dysregulation of lncRNAs is closely associated with tumor development. Their roles in processes such as pyroptosis, tumor immunity, and tumor microenvironment have garnered significant attention (23). During pyroptosis, immune components within the tumor microenvironment exert regulatory effects, often by modulating immune cell function (24). Our study on lncRNAs related to pyroptosis and immunity in BC aimed to uncover new therapeutic targets. We developed a lncRNA signature to assess patient outcomes and immunotherapy reactions.

## 2 Materials and methods

### 2.1 Data collection and analysis

BC RNA-Seq data were sourced from the University of California, Santa Cruz (UCSC) Xena Project (https://xena.ucsc.edu/), including datasets from The Cancer Genome Atlas (TCGA) and the Genotype-Tissue Expression (GTEx) project, RSEM expected_count data from TCGA and GTEx, and RSEM transcript per million (TPM) data from TCGA (25). The UCSC Xena project addresses issues, such as limited normal samples in TCGA, improved compatibility between datasets, and standardized raw expression data to minimize variation between sources (26). We converted TCGA and GTEx expected count data from log2 (expected count + 1) data, referred to as TCGA-GTEx expected_count. Similarly, TCGA TPM data were transformed from log2 (TPM + 0.001) values. The TCGA dataset included 1,086 female BC cases and 112 tumor-adjacent normal tissue samples, whereas the GTEx dataset included 79 normal female breast tissue samples. The corresponding patients’ clinical information was collected via a gdc-client.

### 2.2 Identification of pyroptosis-immune-related lncRNAs

Differential gene expression was analyzed using TCGA-GTEx expected count. Annotation of lncRNAs and mRNAs in the dataset TCGA-GTEx was based on the ENSEMBL human gene annotation file (https://ftp.ensembl.org//pub/release-110/gtf/homo_sapiens/). Duplicate genes were removed, retaining only genes with the highest expression. Differentially expressed genes (DEGs) were unveiled using “limma” (v.3.58.1), “edgER” (v.4.0.16), and “DESeq2” (v.1.42.1) with a false discovery rate (FDR) < 0.05 and |log_2_fold change (FC)| ≥ 1. The converging results from these three methods were used to identify the DEGs.

We retrieved 405 pyroptosis-related genes from GeneCards (https://www.genecards.org/) with a relevance score > 1 and acquired 2,483 immune-related genes from the ImmPort database (http://www.immport.org). Using the “limma” package, we calculated and uncovered pyroptosis- and immune-related differentially expressed lncRNAs (DE-PyrolncRNAs and DE-ImmlncRNAs, respectively) using |Pearson’s correlation coefficient| > 0.4| and p-value < 0.01 as the threshold. The final set of PyroI-mm-lncRNAs was obtained by overlapping the DE-pyrolncRNAs and DE-immune-lncRNAs.

### 2.3 Construction of PyroImm-lncRNAs prognostic signatures

BC TCGA TPM data were integrated with prognostic data, focusing solely on overall survival (OS) as the survival endpoint. Using the createDataPartition() function from the R caret package (27), The dataset was assigned to the training and testing subsets to create a prognostic risk model using the createDataPartition function in the R caret package (27). In the training subset, univariate Cox proportional hazards regression (R package library “survival”) identified PyroImm-lncRNAs associated with OS, including genes with P < 0.05. To refine the model and address overfitting, least absolute shrinkage and selection operator (LASSO) Cox proportional risk regression was conducted with parameters family = “cox” and maximum = 1,000 via the “glmnet” package in R (28) to reduce gene numbers. Stepwise Cox regression was then applied to optimize the model (29), calculating each patient’s risk score as the sum of each RNA expression (EXP) multiplied by its coefficient; that is, the formula was as follows: risk score = EXPa × coefficient a + EXPb × coefficient b + EXPc × coefficient c +…+ EXPn × coefficient n, where n is the number of RNAs. Based on these scores, patients were classified into high-risk (HR) and low-risk (LR) groups. Kaplan-Meier (K-M) survival curves (using R packages “survival” and “survivor”) compared OS between these groups in the full, training, and testing datasets, with a log-rank P<0.05 as significance. Receiver operating characteristic (ROC) curves (using R packages “timeROC,” “survival,” and “survivor”) assessed sensitivity and specificity at 1-, 3-, and 5-year OS, while risk heat maps, risk curves, and survival-status maps illustrated the model’s prognostic performance.

### 2.4 Clinicopathological features and nomogram establishment

Univariate and multivariate Cox regression analyses were performed to identify independent prognostic indicators using the R survival package, based on the risk scores of clinicopathological factors, including age, distant metastasis (M) (ajcc_pathology M), lymph node metastasis (N) (ajcc_pathology N), stage (ajcc_pathology stage), estrogen receptor (ER), progesterone receptor (PR), human epidermal growth factor receptor 2 (HER2), and tumor size and invasiveness (T) (ajcc_pathology T). Significance was set at P<0.05, with OS as the endpoint. Stepwise Cox regression (R package “My.stepwise”) refined these factors to create an optimal nomogram model (30). The prognostic performance of the model was examined using the consistency index (C-index), receiver operating characteristic (ROC) curve, and the calibration curve of the full dataset. Decision curve analysis (DCA) with R packages “survival” and “stdca. R” validated the clinical utility of the model, with P<0.05 indicating significance.

### 2.5 Immune landscape analysis

Stromal, immune, and ESTIMATE scores, and tumor purity in each TCGA-BRCA case were evaluated using the R package ESTIMATE (v.1.0.13) (31) and compared across risk groups using the Wilcoxon test. Differences in various immune cells from TCGA database were examined across TIMER, CIBERSORT, CIBERSORT ABS, QUANTISEQ, MCPCOUNTER, XCELL, and EPIC platforms based on their immune scores downloaded from TIMER2.0 (http://timer.compgenomics.org) (32). Immune function scores of thirteen gene sets from TCGA-BRCA (33) were gathered from https://www.ncbi.nlm.nih.gov/pmc/articles/PMC6310928/bin/ and measured using single-sample gene set enrichment analysis (ssGSEA) with R packages “GSEABase” (v.1.64.0), “limma” (v.3.58.1), and “GSVA” (v.1.50.5). Differences between the HR and LR groups were compared using Wilcoxon test. In addition, seventy-nine immune checkpoint genes were downloaded (34), and differences between the HR and LR groups were evaluated using the Wilcoxon test.

### 2.6 GSEA and gene set variation analysis

DEGs were uncovered using R package “limma” (v.3.58.1) based on TCGA expected_count, applying the cut-off threshold of |log_2_FC|> 0.585 and FDR < 0.05. To explore downstream pathways potentially influenced by the signature, three gene sets (“h.all.v2024.1. Hs.symbols”, “c2.cp.kegg_legacy.v2024.1. Hs.symbols”, and “c7.immunesigdb.v2024.1. Hs.symbols”) from the MSigDB database (https://www.gseamsigdb.org/gsea/msigdb/) were examined using GSEA via the R package “GseaVis” (v.0.0.5), and the differences with normalized enrichment scores |NES|>1, P value <0.05, and p.adjust (Method = ‘Benjamini and Hochberg’) <0.05 were considered statistically significant. For GSVA, two gene sets (“h.all.v2024.1. Hs.symbols” and “c2.cp.kegg_legacy.v2024.1. Hs.symbols”) from the MSigDB (https://www.gseamsigdb.org/gsea/msigdb/) were selected. Their GSVA scores in HR and LR groups were calculated and compared using R packages “GSEABase” (v.1.64.0), “limma” (v.3.58.1), and “GSVA” (v.1.50.5) to unveil differentially enriched functions and pathways with the threshold of a GSVA score |t-value| >2 (35).

### 2.7 Validation of PyroImm-lncRNA signature by single nuclear RNA-seq data

The GSE176078 dataset from the GEO database (https://www.ncbi.nlm.nih.gov/geo/) (36) was used to validate the PyroImm-lncRNA signature using the R package Seurat (v.5.1.0, https://Github/Satijalab/Seurat). The number of genes and mitochondrial gene proportions in each cell line were determined. Cells expressing < 200 genes or genes expressed in < three cells were excluded. After quality control using the R package basic_qc, qualified snRNA seq data was normalized using the “LogNormalize” method. Principal Component Analysis (PCA) and Uniform Manifold Estimation and Projection (UMAP) were applied to minimize the size.

### 2.8 Statistical evaluation

All evaluations were performed using R v.4.3.1. Classification variables were reported as counts and percentages. Quantitative comparisons were made using the Wilcoxon rank-sum test, and categorical comparisons were made using the Chi-square test. Linear correlations were inspected using Pearson’s correlation with significance set at P<0.05.

## 3 Results

### 3.1 Examination of PyroImm-lncRNAs

**Figure 1:**
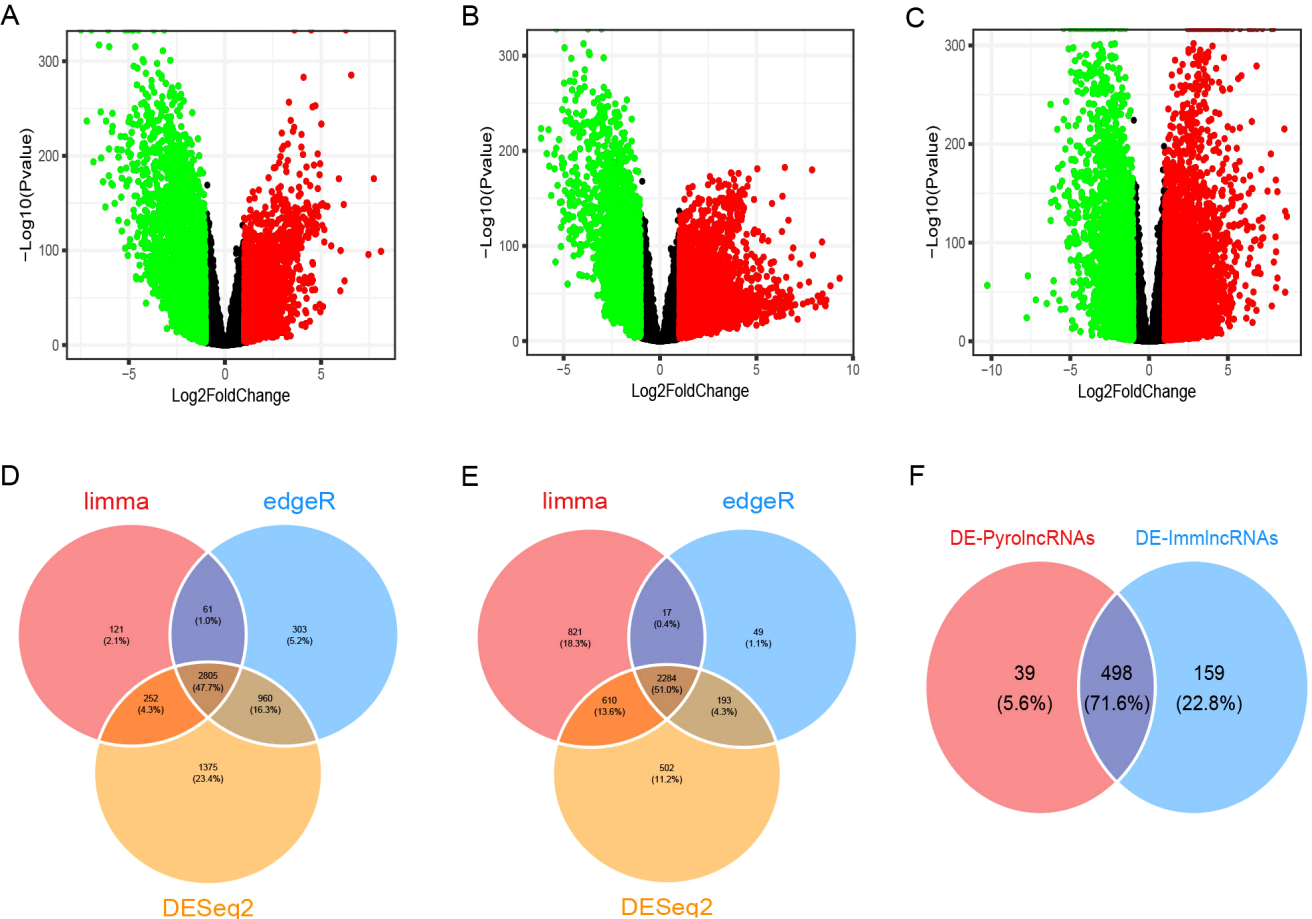
Identification of differentially expressed genes (DEGs) and pyroptosis-immune-related long non-coding RNAs (lncRNAs) in breast cancer. **(A-C)** The volcano plots of DEGs analyzed using the limma, edgeR, and DESeq2 methods, respectively. **(D, E)** The Venn diagram shows the overlap of upregulated and downregulated DEGs. **(F)** The Venn diagram shows the overlap of the DE-PyrolncRNAs and DE-ImmlncRNAs.DE-PyrolncRNAs, the pyroptosis-related differentially expressed lncRNAs; DE-ImmlncRNAs, immune-related differentially expressed lncRNAs.

A total of 33,540 genes, including mRNAs and lncRNAs, were extracted from the TCGA-GTEx expected_count. DEGs between BC and normal tissues were unveiled using the R packages “limma,” “edgeR,” and “DESeq2,” with overlapping DEGs from the three methods selected. In total, 5,089 DEGs were identified, including 2,805 upregulated and 2,284 downregulated genes, as shown in the volcano plots (**Figures 1A–E**). Among these, 1,180 were differentially expressed lncRNAs (DE-lncRNAs), comprising 724 upregulated and 456 downregulated lncRNAs. Pearson’s correlation analysis of DE-lncRNAs with pyroptosis-related genes and immune-related genes identified 537 DE-pyrolncRNAs and 657 DE-immune-related genes. After overlapping the DE-pyrolncRNAs and DE-ImmlncRNAs, 498 PyroImm-lncRNAs were identified (**Figure 1F**).

### 3.2 Prognostic risk model construction

**Figure 2:**
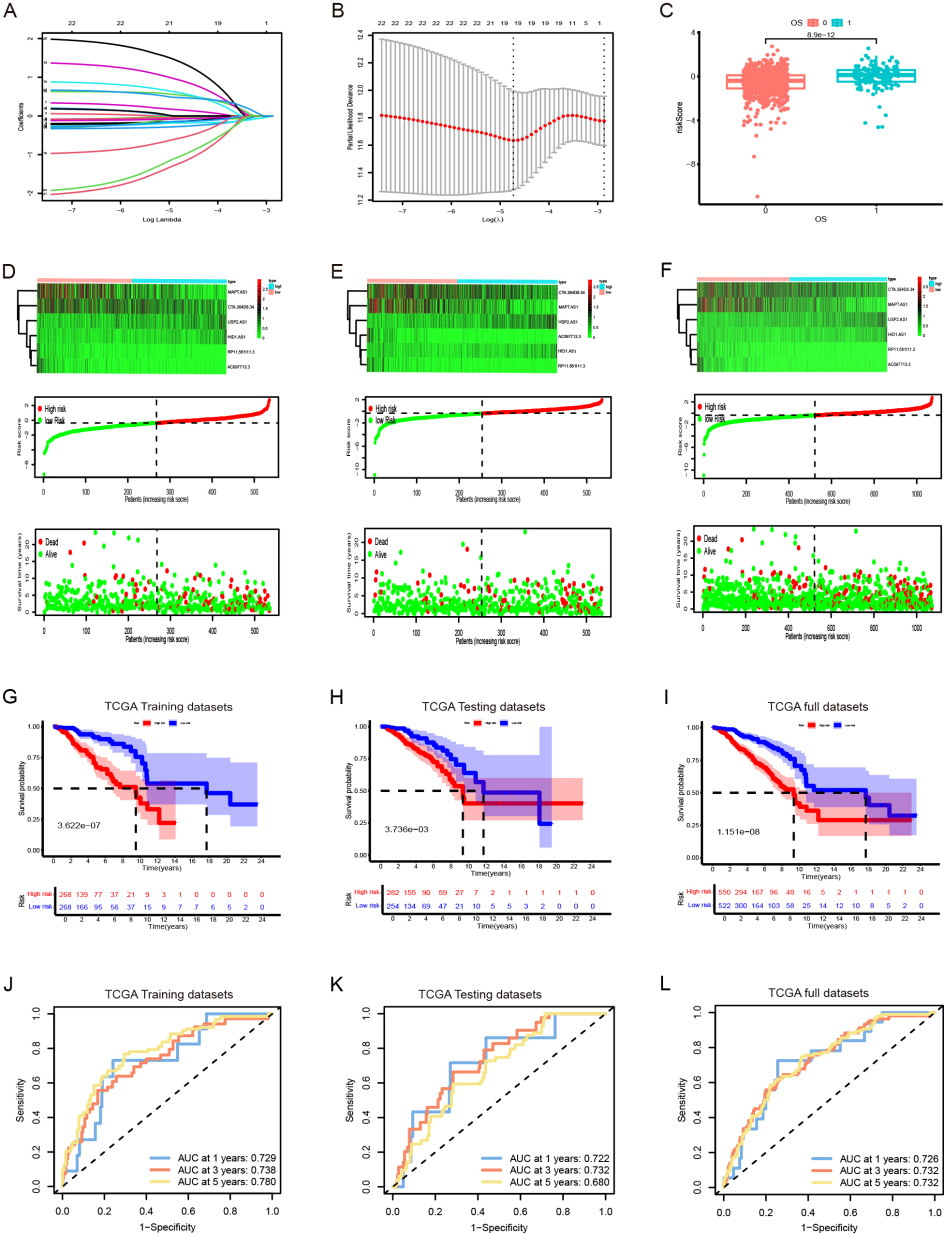
The development and validation of prognostic signatures for pyroptosis-immune-related lncRNAs (PyroImm-lncRNAs). **(A)**The distribution of coefficient in the LASSO regression analysis. **(B)** the distribution of lambda values in the LASSO regression analysis. **(C)** Comparison of risk scores among patients with different overall survival (OS). **(D–F)** RNA expression heatmap(top), Plot of risk score(middle), and survival status (below) of patients in the training datasets、testing datasets and full datasets respectively. **(G–I)** Kaplan-Meier survival analyses for high- and low-risk patients across training, testing, and full datasets respectively. **(J–L)** Receiver operating characteristic (ROC) curves evaluating the predictive efficacy of the signature for patients’ 1-year, 3-year, and 5-year survival rates in the training datasets、testing datasets and full datasets respectedly. LASSO, least absolute shrinkage and selection operator; OS, overall survival; TCGA, The Cancer Genome Atlas.

TCGA TPM data were merged with the downloaded clinicopathological data, and OS was used as the sole survival target, resulting in 1,072 patients. Using the createDataPartition() function from the R caret package, the dataset was assigned to training and testing subsets in a ratio of 5:5. Univariate Cox proportional hazards regression examination of the training subset via the R library “survival” package identified PyroImm-lncRNAs linked to OS prognosis, with genes showing P < 0.05 selected. To reduce the number of lncRNAs and address overfitting, LASSO Cox regression analysis further refined the model to 19 genes (**Figures 2A, B**; **Table 1**). Stepwise Cox regression was used to derive an optimal six-lncRNA prognostic model, including MAPT.AS1, CTA.384, D8.34, RP11.561, I11.3, HID1.AS1, AC097713.3, and USP2.AS1 (**Table 2**), with a concordance index of 0.743 (se = 0.031, p=2e-09). Based on the median risk score of the training subset, the cases were assigned to the HR and LR groups. HR scores correlated with mortality (P = 8.9e-12) (**Figure 2C**), and risk heat maps, risk curves, and survival status maps were created using pHeat maps (**Figures 2D–F**). The K-M survival curves revealed a substantially poorer prognosis for the HR group across the training, testing, and full datasets (P = 3.622e-07, P = 3.736e-03, and P = 1.151e-08, respectively) (**Figures 2G–I**). The ROC curves demonstrated predictive values for the model, with areas under the ROC curve (AUCs) of 0.729, 0.738, and 0.780 for 1-, 3-, and 5-year OS in the training subset, 0.722, 0.732, and 0.680 in the testing subset, and 0.726, 0.732, and 0.732 in the full dataset (**Figures 2J–L**), indicating that the six lncRNA prognostic models had predictive values for OS.

**Table 1:** Pyroptosis-immune-related lncRNAs related to overall survival (OS) in the training set.

id	UniCox				LASSO
	HR	HR.95L	HR.95H	p-value	coefficient
RP11-161H23.5	0.828	0.691	0.991	0.0399^*^	-0.139
MIR205HG	0.858	0.771	0.955	0.0050^**^	
RP11-638I2.6	0.736	0.596	0.908	0.0043^**^	-0.011
LIPE-AS1	0.761	0.613	0.944	0.0128^*^	
RP11-1143G9.4	0.868	0.764	0.985	0.0286^*^	-0.069
RP11-303E16.2	1.368	1.059	1.767	0.0166^*^	
PDXDC2P	1.454	1.014	2.085	0.0419^*^	0.154
RP11-459E5.1	0.817	0.692	0.965	0.0176^*^	-0.140
RP11-265N6.1	0.799	0.658	0.972	0.0246^*^	-0.035
LINC00853	0.666	0.456	0.971	0.0346^*^	-0.062
CTA-384D8.34	0.769	0.600	0.985	0.0377^*^	-0.269
MAPT-AS1	0.730	0.600	0.888	0.0017^**^	-0.179
CTD-2589H19.6	0.541	0.315	0.930	0.0263^*^	-0.071
GRIK1-AS1	0.736	0.543	0.996	0.0470^*^	
RBM5-AS1	0.548	0.301	0.999	0.0495^*^	-0.558
RP11-290O12.2	1.862	1.112	3.118	0.0181^*^	0.536
ZEB2-AS1	0.344	0.128	0.926	0.0347^*^	-0.137
USP2-AS1	1.786	1.055	3.023	0.0310^*^	0.586
C9orf163	2.125	1.060	4.261	0.0336^*^	0.907
HID1-AS1	2.594	1.083	6.211	0.0324^*^	1.372
RP11-561I11.3	0.203	0.042	0.990	0.0485^*^	-1.193
AC097713.3	0.247	0.068	0.900	0.0340^*^	-1.018
RP11-439A17.9	2.027	1.167	3.522	0.0121^*^	0.472

UniCox, univariate Cox analysis; HR, Hazard Ratio; LASSO, least absolute shrinkage and selection operator; *:<0.05; **:<0.01.

**Table 2:** Stepwise regression screening for the optimal prognostic model.

	coefficient	HR	HR.95L	HR.95H	p-value
MAPT.AS1	-0.386	0.680	0.556	0.832	0.000179 ^***^
CTA.384D8.34	-0.421	0.656	0.506	0.851	0.001452 ^**^
RP11.561I11.3	-2.000	0.136	0.029	0.633	0.010996 ^*^
HID1.AS1	1.883	6.575	2.489	17.368	0.000145 ^***^
AC097713.3	-1.737	0.176	0.041	0.764	0.020372 ^*^
USP2.AS1	0.770	2.161	1.248	3.740	0.005937 ^**^

HR, Hazard ratio; ***, 0.001; **, 0.01; *, 0.05.

### 3.3 Nomogram model establishment and validation

**Figure 3:**
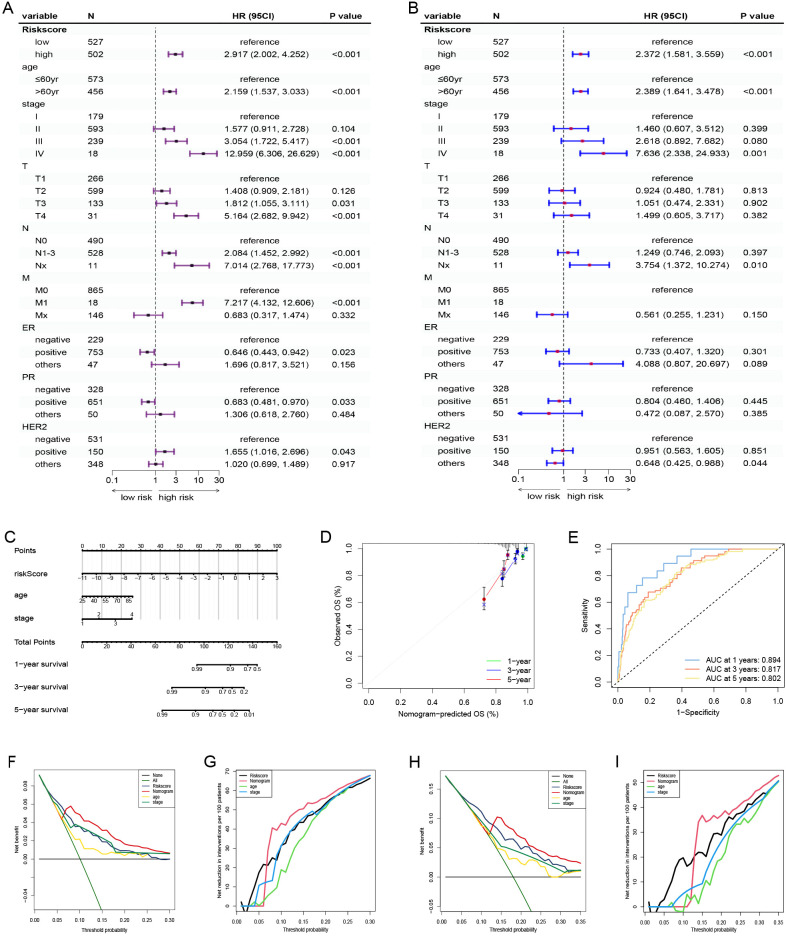
Nomogram model establishment and validation. **(A)** Univariate and **(B)** Multivariate Cox regression analysis of clinical factors and risk score. **(C)** A nomogram established in the entire cohort by combining risk score and other clinical factors containing age, staging. **(D)** Calibration curve used to assess the agreement between nomogram-predicted survival and true survival of patients in the entire cohort. **(E)** ROC curve for assessing the efficacy of the Nomogram in predicting patients’ 1-year, 3-year, 5-year survival rates. **(F–I)** DCA comparing the efficacy of nomogram, risk score, age and staging in predicting patients’ 3-year and 5-year survival rates. M, ajcc_pathology M; N, ajcc_pathology N, staging, ajcc_pathology stage; T, ajcc_pathology T; ROC, Receiver operating characteristic; DCA, Decision curve analysis.

Based on the survival analysis, 1029 patients with BC with detailed clinicopathological characteristics were included. The key variables were the risk score, age, distant metastasis (M) (ajcc_pathology M), lymph node metastasis (N) (ajcc_pathology N), staging (ajcc_pathology staging), tumor size and invasiveness (T) (ajcc_pathology T), ER,PR,HER2,and mortality (**Table 3**). The HR group displayed a significantly elevated risk score, advanced staging, advanced T,negative estrogen receptor (ER) status, negative progesterone receptor (PR) status, positive human epidermal growth factor receptor 2 (HER2) status,and mortality compared to the LR group (P<0.05), while other clinical features showed no significant differences. Univariate analysis revealed that age, stage(III vs I;IV vs I), T(T3 vs T1;T4 vs T1), N(N1-3 vs N0;Nx vs N0),M(M1vsM0),ER(positive vs negative),PR(positive vs negative), HER2(positive vs negative), and risk score were prognostic factors (P<0.05) (**Figure 3A**), whereas multivariate regression analysis highlighted age, stage(IV vs I), N(Nx vs N0),HER2 (others vs negative),and risk score as independent prognostic factors (P<0.05) (**Figure 3B**). An optimal nomogram model was developed using stepwise Cox regression (R package “My.stepwise”) and constructed with R package “survival (v3.3-1)”, incorporating age, stage, and risk score (**Table 4**). This model showed strong predictive performance, with a concordance index of 0.801 (SE = 0.019, P=2e-16). The nomogram was visualized using R package “survival (v3.3-1)” “rms,” and “survivcomp” (**Figure 3C**), and calibration curves indicated accurate 1- and 3-year OS predictions, though 5-year predictions were slightly less accurate (**Figure 3D**). The ROC curves demonstrated robust prognostic values across 1-, 3-, and 5-year prognoses with all AUC exceeding 0.8, and the full dataset C-index was 0.801 (CI: 0.68–0.88) (**Figure 3E**). Additionally, DCA curves confirmed the clinical effectiveness of both the nomogram and the risk models, with the nomogram model showing superior net benefits at the 3- and 5-year time points (**Figures 3F–I**).

**Table 3:** Clinical pathological features of patients between high- and low-risk groups.

	Overall (1029)	Low-risk (426)	High-risk (465)	P value
Risk score (median [IQR])				
	-0.34[-1.62,0.78]	-1.05[-2.0,-0.21]	0.17[-0.43,0.88]	<0.001
Age (median [IQR])				
	58[40,76]	57[37.9,75.4]	59[41,77]	0.456
ajcc_pathology T(%)				0.002
T1	266 (25.85%)	152 (30.28%)	114 (21.63%)	
T2	599 (58.21%)	273 (54.38%)	326 (61.86%)	
T3	133 (12.93%)	68 (13.55%)	65 (12.33%)	
T4	31 (3.01%)	9 (1.79%)	22 (4.17%)	
ajcc_pathology N(%)				0.805
N0	490 (47.62%)	243 (48.41%)	247 (46.87%)	
N1-3	528 (51.31%)	253 (50.40%)	275 (52.18%)	
Nx	11 (1.07%)	6 (1.20%)	5 (0.95%)	
ajcc_pathology M(%)				0.429
M0	865 (84.06%)	418 (83.27%)	447 (84.82%)	
M1	18 (1.75%)	7 (1.39%)	11 (2.09%)	
Mx	146 (14.19%)	77 (15.34%)	69 (13.09%)	
ajcc_pathology stage(%)				0.016
I	179 (17.40%)	103 (20.52%)	76 (14.42%)	
II	593 (57.63%)	291 (57.97%)	302 (57.31%)	
III	239 (23.23%)	101 (20.12%)	138 (26.19%)	
IV	18 (1.75%)	7 (1.39%)	11 (2.09%)	
ER:				<0.001
Negative	229 (22.25%)	77 (15.34%)	152 (28.84%)	
Positive	753 (73.18%)	400 (79.68%)	353 (66.98%)	
others	47 (4.57%)	25 (4.98%)	22 (4.17%)	
PR:				<0.001
Negative	328 (31.88%)	111 (22.11%)	217 (41.18%)	
Positive	651 (63.27%)	365 (72.71%)	286 (54.27%)	
others	50 (4.86%)	26 (5.18%)	24 (4.55%)	
HER2:				0.001
Negative	531 (51.60%)	274 (54.58%)	257 (48.77%)	
Positive	150 (14.58%)	52 (10.36%)	98 (18.60%)	
others	348 (33.82%)	176 (35.06%)	172 (32.64%)	
Vital status (%)				<0.001
Alive	891 (86.59%)	464 (92.43%)	427 (81.02%)	
Dead	138 (13.41%)	38 (7.57%)	100 (18.98%)	

IQR, interquartile range; ER, estrogen receptor; PR: progesterone receptor; HER2, human epidermal growth factor receptor 2.

**Table 4:** Stepwise regression screening for the optimal nomogram model.

	coefficient	HR	HR.95L	HR.95H	pvalue
riskScore	0.643	1.901	1.545	2.341	1.35×10^-9 ***^
TMN	0.710	2.034	1.621	2.552	8.89×10^-10 ***^
age	0.794	2.212	1.574	3.110	4.92×10^-6 ***^

HR, Hazard ratio; ***:0.001.

### 3.4 Partial validation of prognostic risk model

**Figure 4:**
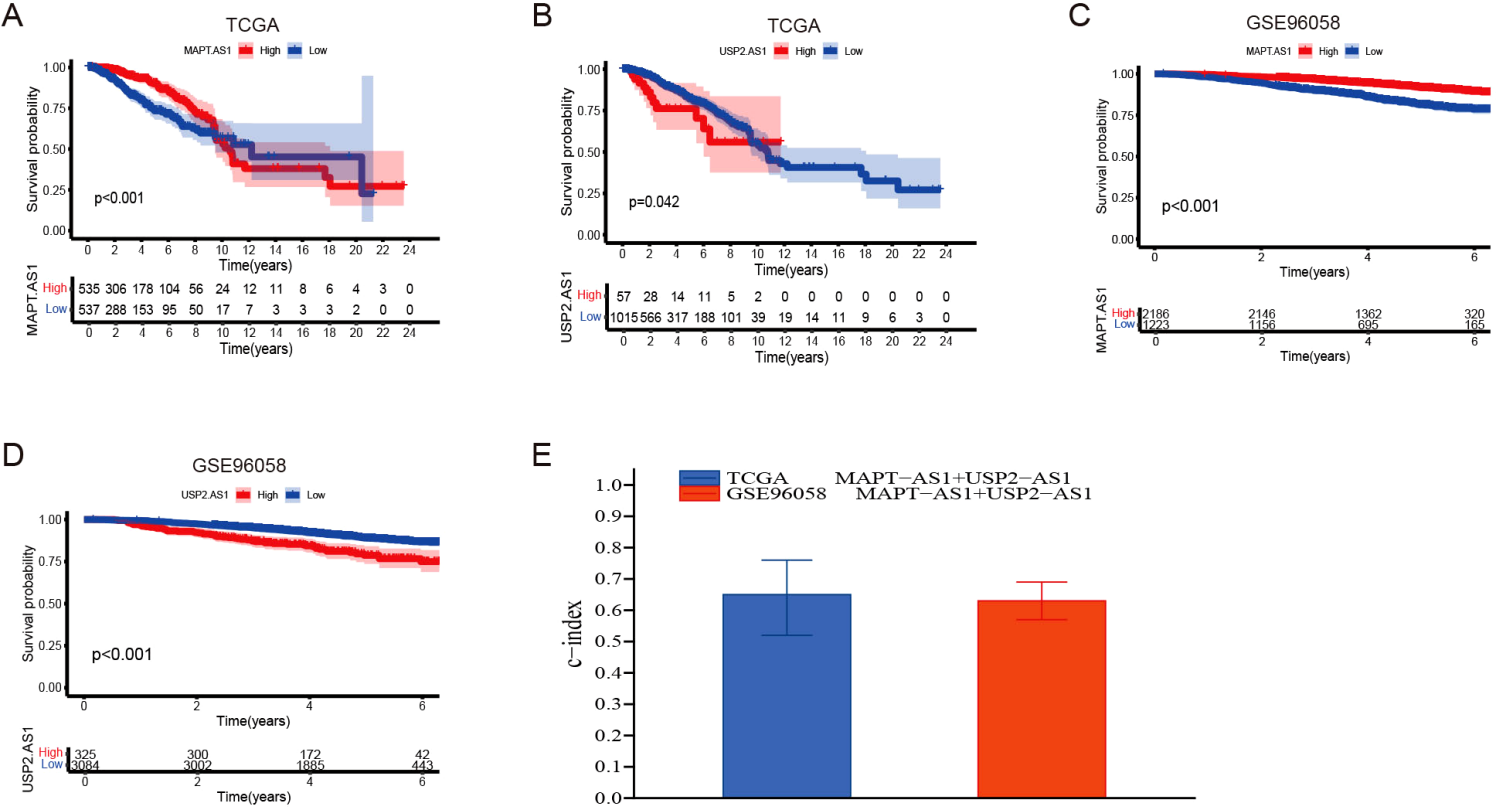
Partial Validation of prognostic risk model. **(A, B)** Kaplan-Meier survival analyses were performed for patients with MAPT-AS1 and USP2-AS1, employing data obtained from The Cancer Genome Atlas (TCGA) datasets. **(C, D)** Kaplan-Meier survival analyses were performed for patients with MAPT-AS1 and USP2-AS1, employing data obtained from the GSE96058 dataset. **(E)** Evaluate the collective prognostic predictive abilities of MAPT-AS1 and USP2-AS1 within the TCGA and GSE96058 datasets.

The dataset utilized in this study was sourced from the Gene Expression Omnibus (GEO) under the accession number GSE96058 (platform GPL11154), which includes a total of 3,069 breast cancer (BC) cases. The X-tile software was employed to identify the optimal threshold value, and subsequent Kaplan-Meier analysis indicated that the genes MAPT-AS1 and USP2-AS1 possess significant prognostic relevance within both the GSE96058 dataset and the TCGA dataset (**Figures 4A–D**). To assess the combined prognostic prediction capabilities of MAPT-AS1 and USP2-AS1, the concordance index (C-index) was calculated. The C-index for the GSE96058 dataset was found to be 0.633 (95% CI: 0.57, 0.69), while the C-index for the TCGA dataset was determined to be 0.647 (95% CI: 0.52, 0.76) (**Figure 4E**).

**Figure 5:**
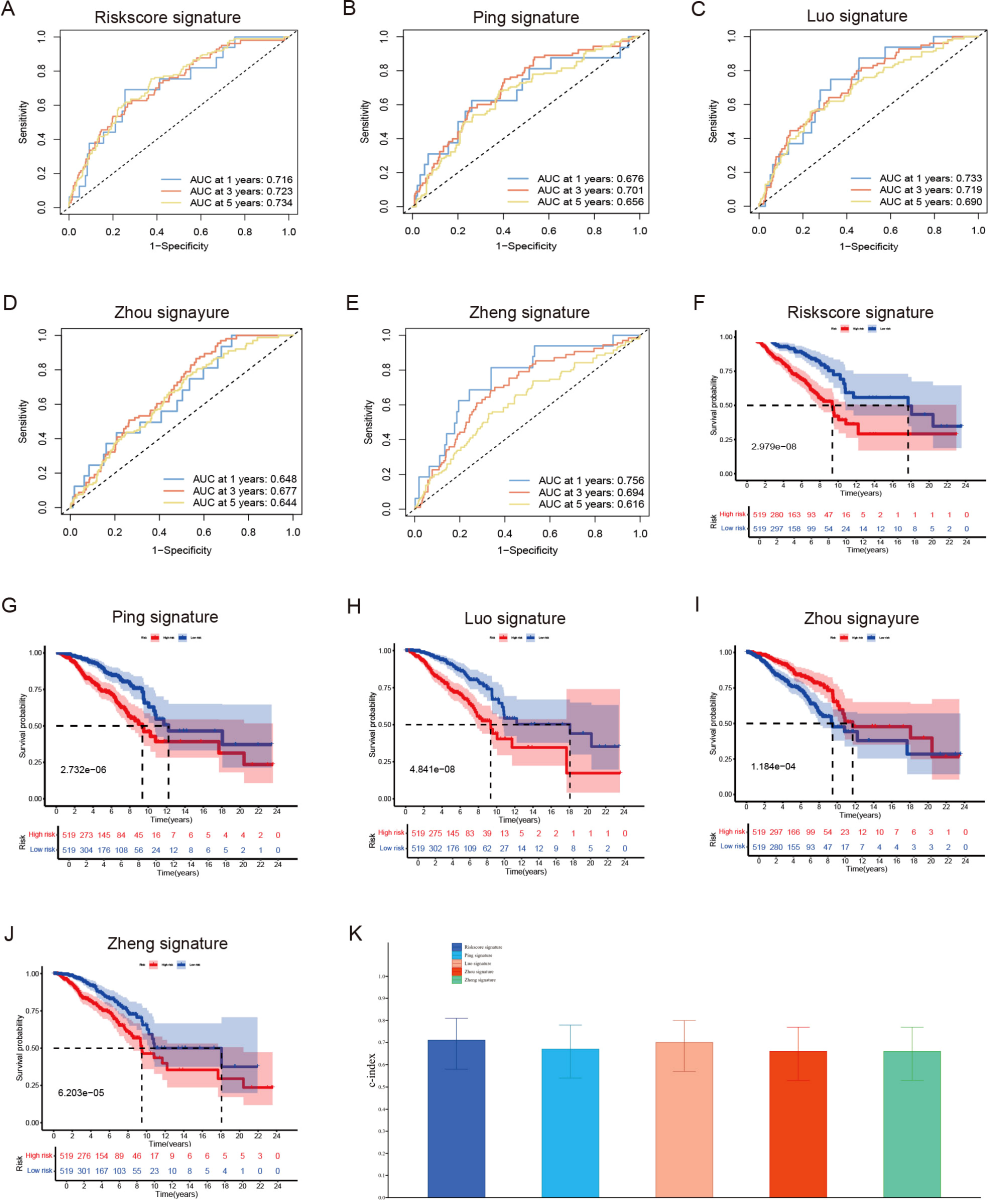
Evaluate the predictive validity of different prognostic models. **(A–E)** Univariate and **(B)** Multivariate Cox regression analysis of clinical factors and risk score. **(F–J)** ROC curve for assessing the efficacy of different prognostic models in predicting patients’ 1-year, 3-year, 5-year survival rates. **(K)** The consistency index demonstrates the reliability of our six lncRNA model when compared to other prognostic models.

By synthesizing data acquired from the TCGA data portal (https://portal.gdc.cancer.gov/) with our results from the UCSC Xena Project (https://xena.ucsc.edu/), we conducted a prognostic analysis involving 1,038 female breast cancer patients to assess the predictive accuracy of various prognostic models. The area under the curve (AUC) values for 1-year, 3-year, and 5-year survival rates all surpassed 0.7. This finding indicates that our novel model, which integrates six long non-coding RNAs (lncRNAs), surpasses previously established prognostic signatures developed by researchers such as Ping et al. (37), Luo et al. (38), Zhou et al. (39), and Zheng et al. (40) (**Figures 5A–E**). Additionally, the Kaplan-Meier analysis demonstrated significant disparities in overall survival rates between low- and high-risk breast cancer patients as categorized by our risk signature and other models (**Figures 5F–J**). Importantly, our risk model exhibited the highest consistency index, thereby bolstering the reliability of our six lncRNA model in predicting breast cancer outcomes (**Figure 5K**).

### 3.5 Immune landscape analysis

**Figure 6:**
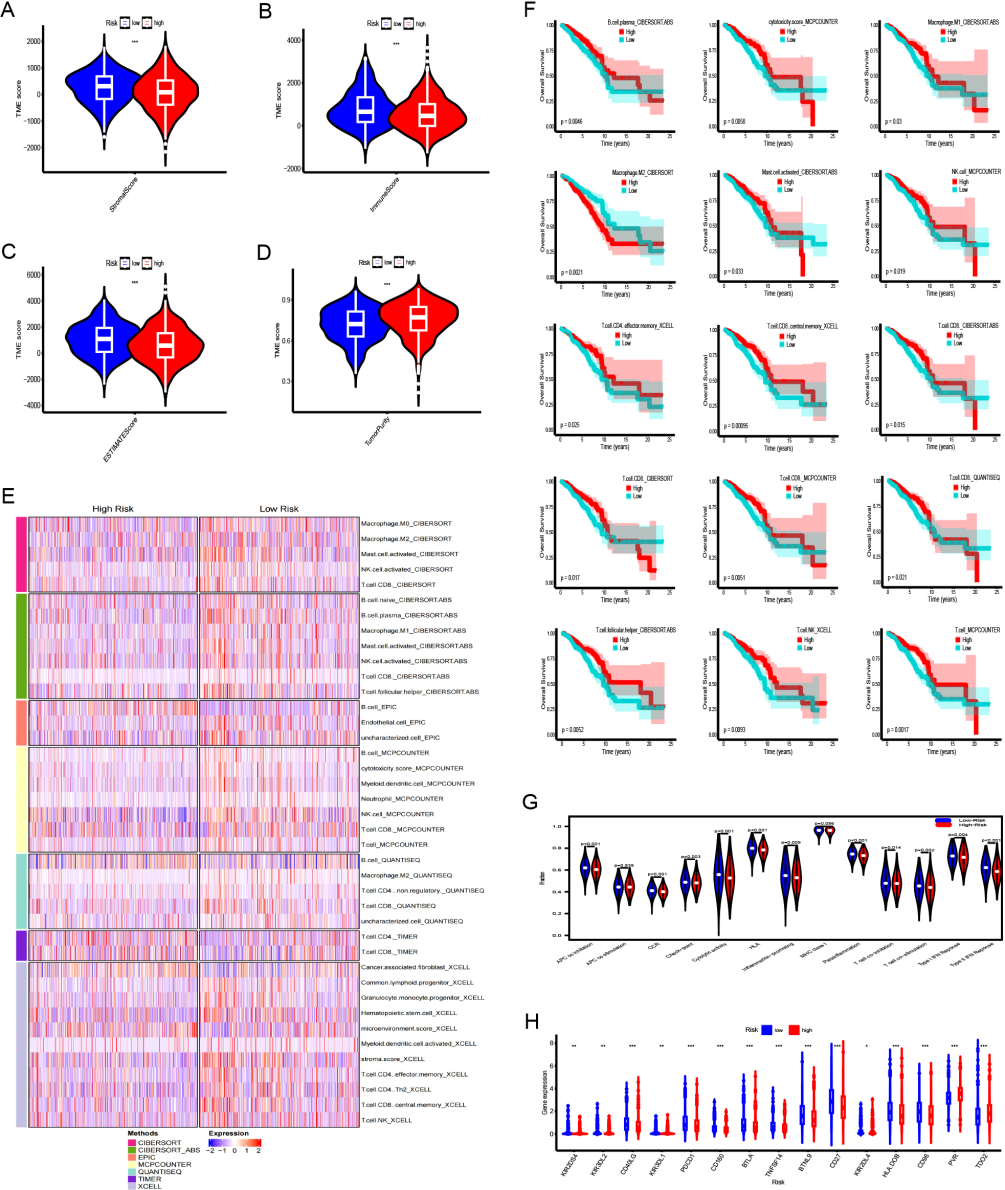
Immune landscape analysis between the high-risk (HR) and low-risk (LR) groups. **(A–D)** Comparison of stromal scores, immune scores, ESTIMATE scores, and tumor purity for tumors in both HR and LR groups. **(E)** Heatmap illustrating differences in immune cell infiltration between HR and LR groups. **(F)** The Kaplan-Meier curve comparing the effects of these immune cell infiltrations on the survival of breast cancer patients. **(G)** Differences in immune functions between high- and low-risk groups. **(H)** Differences in expression of immune checkpoints between high- and low-risk groups. ***:0.001;**:0.01;*:0.05.

Stromal, immune, and ESTIMATE scores were increased in the LR group (**Figures 6A–C**), whereas tumor purity was notably elevated in the HR group (**Figure 6D**). The TIMER, CIBERSORT, CIBERSORT-ABS, QUANTISEQ, MCPCOUNTER, XCELL, and EPIC platforms were used to investigate immune cell infiltration in relation to risk scores. Across these platforms, 58 immune cells showed significant differences between groups (P < 0.05) (**Figure 6E**). K-M survival analysis further revealed that 15 of these immune cells correlated with distinct prognoses depending on their expression levels (**Figure 6F**). Next, we examined the association between risk scores, immune-related functions, and immune checkpoints. Using ssGSEA, we assessed immune function scores in TCGA-BRCA samples and found significant differences in 11 immune functions (**Figure 6G**). Functions such as APC co-inhibition, CCR, checkpoints, cytolytic activity, HLA, inflammation promotion, para-inflammation, T-cell co-inhibition, T-cell co-stimulation, type I IFN response, and MHC class I were poorer in the HR group. Further examination of immune checkpoints revealed that 38 immune checkpoints, including KIR2DS4, KIR3DL2, CD40LG, KIR3DL1, and PDCD1, were significantly elevated in the LR group, whereas others such as TDO2, PVR, and CD276 were upregulated in the HR group. Immune checkpoints with log_2_FC ≥0.2 are shown in **Figure 6H**.

### 3.6 GSEA and GSVA

**Figure 7:**
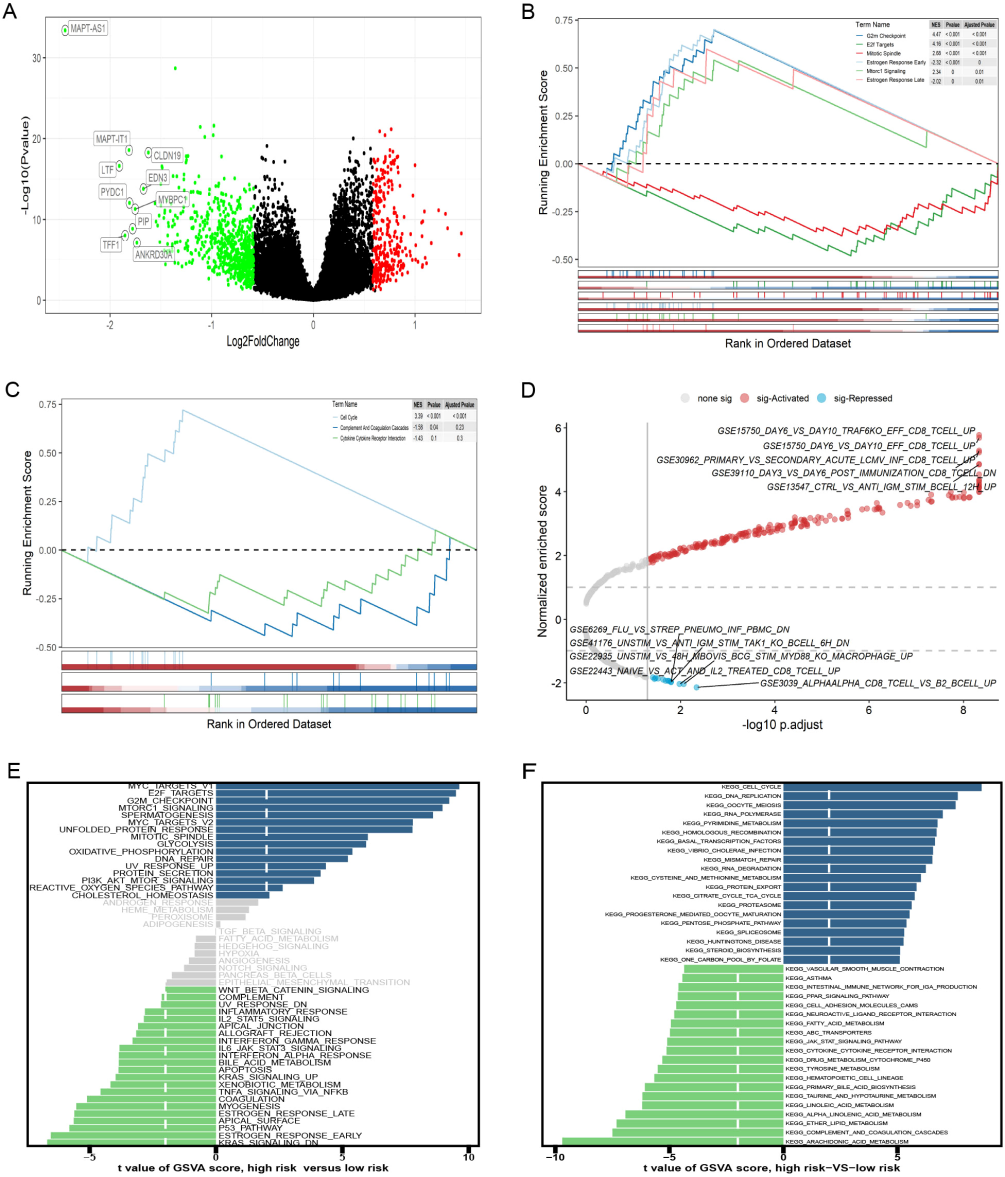
Gene set enrichment analysis (GSEA) and gene set variation analysis (GSVA) of high- and low-risk groups. **(A)** The volcano plot of differentially expressed genes (DEGs). **(B-D)** GSEA results for the gene sets “h.all.v2024.1.Hs.symbols,” “c2.cp.kegg_legacy.v2024.1.Hs.symbols,” and “c7.immunesigdb.v2024.1.Hs.symbols,” respectively. **(E, F)** GSEA results for the gene sets “h.all.v2024.1.Hs.symbols” and “c2.cp.kegg_legacy.v2024.1.Hs.symbols,” respectively.

DEGs across different groups were unveiled using the R package “limma” (v.3.58.1), applying a threshold of |log_2_FC|>0.585 and FDR <0.05. We identified 994 DEGs (**Figure 7A**), 195 lncRNAs, and 799 mRNAs. GSEA pathway analysis of the 799 mRNAs identified significant pathways, including G2M checkpoint, E2f targets, mitotic spindle, estrogen response early, mtorc1 signaling, and estrogen response late, in the “h.all-v2024.1. Hs. (**Figure 7B**). Analysis using the “c2. cp. kegg.legacy. v2024.1. Hs. symbol”gene set was significant only in the cell cycle pathway (**Figure 7C**). GSEA analysis of the gene set of “c7. immunosgdb. v2024.1. Hs. symbols” identified 221 immune gene sets that were significantly enriched; the top five positive and negative absolute enrichment scores are shown in **Figure 7D**. GSVA of “h.all.V2024.1. Hs. symbols” and “c2. cp. kegg.legacy. V2024.1. Hs. symbol” gene sets reveal the most significant pathways. Out of the 50 pathways in “h.all. v2024.1. Hs. symbols,” 37 pathways reached statistical significance with a GSVA score of |t-value| >2 (**Figure 7E**). For “c2. cp. kegg. legacy. v2024.1. Hs. symbols”, 113 out of 186 pathways showed statistically significant differences, with the key pathways displayed in **Figure 7F**.

### 3.7 Validation of PyroImm-lncRNA signatures using snRNA-seq data

**Figure 8:**
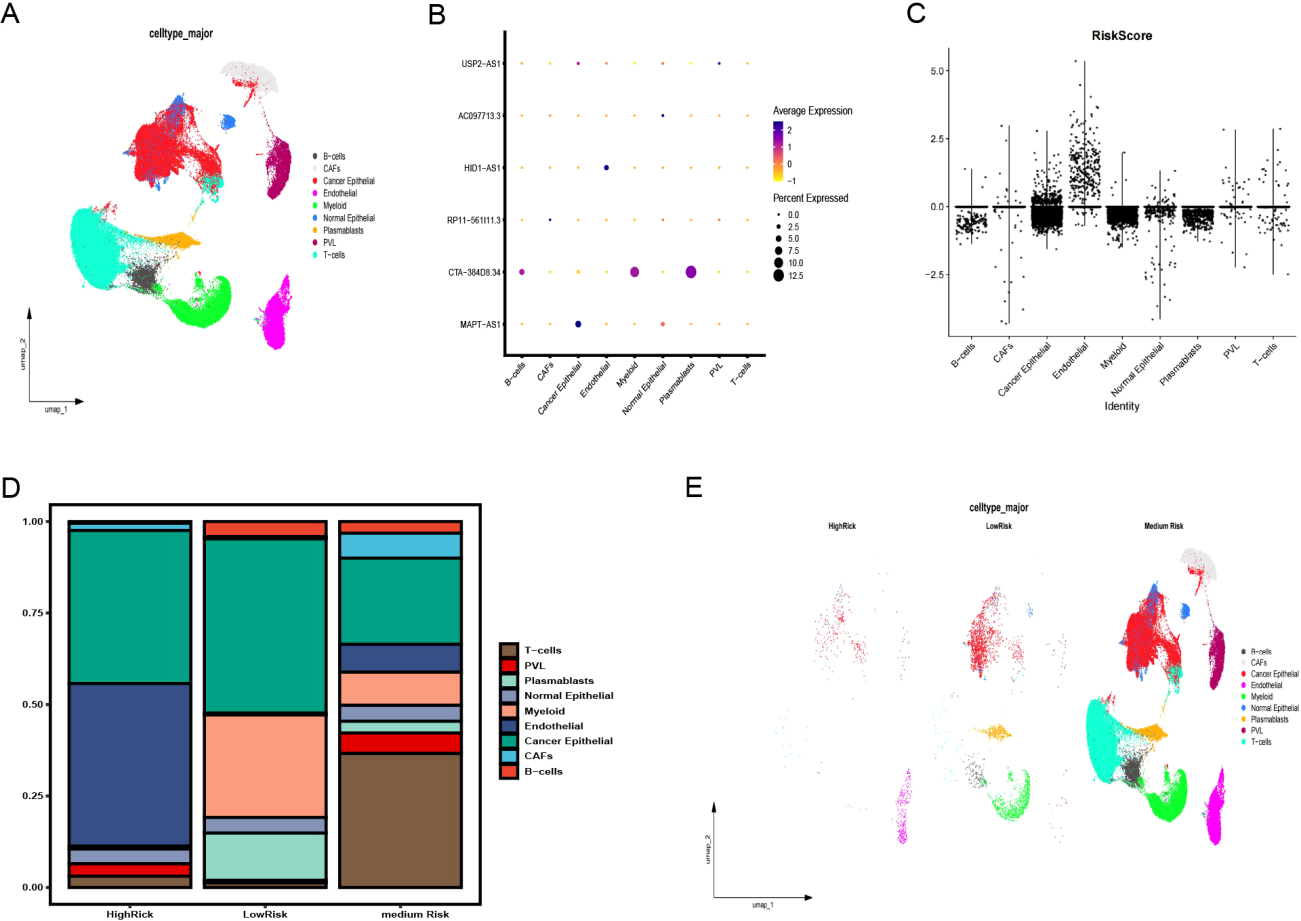
Validation of the pyroptosis-immune-related lncRNA signature using snRNA-seq data. **(A)** Annotation of clusters and UMAP visualization. **(B)** Distribution of six pyroptosis-immune-related signature lncRNAs across each cell type and in all cells. **(C)** Risk scores for each cell type. **(D)** Proportion of each cell type in high-risk (HR), medium-risk (MR), and low-risk (LR) groups. **(E)** Annotation of clusters and UMAP visualization in HR, MR, and LR groups. UMAP, Uniform Manifold Estimation and Projection.

GSE176078 classified cells by type (**Figure 8A**), showing the distribution of six pyroptosis immunity-related signature lncRNAs in each cell type and in all cells (**Figure 8B**). A violin plot of the risk scores, calculated from the coefficients of these six lncRNAs, identified endothelial cells as having the highest risk scores (**Figure 8C**). Depending on the risk scores (>, =, and < 0), the cases were categorized into the HR, medium-risk (MR), and LR groups. Endothelial and cancer epithelial cells predominantly aggregated in the HR group, whereas B cell, myeloid, and plasmablast clusters were concentrated in the LR group and T cell clusters were concentrated in the MR group (**Figures 8D, E**). These distinct cellular aggregations across the different risk groups indicate varying mechanisms of immune evasion.

## 4 Discussion

Growing evidence highlights the importance of pyroptosis genes in cancer progression, where dysregulated pyroptosis fosters tumor growth and development (41–43). Although apoptosis suppresses tumor cell proliferation, invasion, and metastasis, it creates an immunosuppressive microenvironment conducive to tumor expansion (44). Immune cells regulating tumor cell pyroptosis appear to depend on immune cell distribution and subtypes within the tumor, necessitating further research to clarify the regulation of pyroptosis and immune evasion mechanisms in specific tumors (24). In this study, we identified 498 pyroptosis-related lncRNAs in BC samples, from which a prognostic model of six lncRNAs, − MAPT.AS1, CTA.384D8.34, RP11.561I11.3, HID1.AS1, AC097713.3, and USP2. AS1− was developed. Patient risk scores derived from lncRNA expression and associated coefficients revealed significant OS disparities across different groups in the KM survival curves. The HR group consistently exhibited poorer prognosis in the full, training, and test datasets. For the 1- and 3-year prognoses, the ROC curve AUCs exceeded 0.7, whereas the 5-year AUCs reached 0.780, 0.680, and 0.732 for the training, testing, and full datasets, respectively, confirming the prognostic potential of the six-lncRNA model. Stepwise Cox regression analysis of 1029 patients with complete clinicopathological characteristics revealed age, stage, and risk score as prognostic factors, yielding an optimal nomogram model with a concordance of 0.801 (se=0.019 and P=2e-16). This nomogram effectively visualizes survival probabilities and simplifies prediction, displaying a calibration across datasets (45, 46) with a C-index of 0.801 at confidence intervals of (0.68, 0.88) and AUCs > 0.8, for 1-, 3-, and 5-year OS. DCA curves indicated that both the nomogram and risk models provided good net benefits, with the nomogram model displaying superior clinical value.

The six lncRNA prognostic models demonstrated clear predictive value, owing to significant prognostic differences across different risk groups. To analyze immune evasion across these groups, we first evaluated each TCGA-BRCA sample using the ESTIMATE R package. The stromal, immune, and ESTIMATE scores were markedly elevated in the LR group, whereas tumor purity was augmented in the HR group. Immune cell infiltration was explored using seven analytical tools: − TIMER, CIBERSORT, CIBERSORT-ABS, QUANTISEQ, MCPCOUNTER, XCELL, and EPIC. Across the different risk groups, 58 immune cell types showed significant differences (P<0.05). By correlating these 58 immune cells with patient prognosis in the KM survival analysis, we identified a prognostic distinction in the expression of 15 immune cells. Specifically, elevated macrophageM2_CIBERSORT expression in BC correlated with poorer prognosis and was more common in the HR group, whereas 14 other immune cells showed poorer prognosis with low expression in the HR group. These findings revealed the potential of immunotherapy to enhance the prognosis of patients with HR. We then assessed the immune-related functions and found notable differences in 11 immune functions across the different risk groups, with the HR group demonstrating weaker immune functionality. Additionally, 38 immune checkpoint genes were upregulated in the LR group, and three were upregulated in the HR group. These disparities in immune cell profiles, functionality, and checkpoint gene expression indicate distinct immune evasion mechanisms across groups, highlighting the potential of targeted immunotherapy for improving patient outcomes. Pathway differences across the risk groups were analyzed using |log_2_FC |>0.585 and FDR <0.05, and 799 significantly differentially expressed mRNAs were identified. GSEA on the “c7. immunesgdb. v2024.1. Hs. symbols” gene sets revealed 221 significant immune gene sets. Further GSVA on “h.all. v2024.1. Hs. symbols” and “c2. cp. kegg. legacy. v2024.1. Hs. Symbols” gene sets showed 37 out of 50 pathways in “h.all. v2024.1. Hs. symbols” and 113 out of 186 pathways in “c2. cp. kegg. legacy. v2024.1. Hs. symbols” were significant, illustrating the distinct biological characteristics of the HR and LR groups. Finally, we validated the pyroptosis-related lncRNA signature using snRNA-seq data (GSE176078) with clusters based on the cell type _major metadata. The HR group possessed very few T cells, B cells, myeloids, and plasmablast clusters, whereas the LR group displayed significant B-cells, myeloids, and plasmablast clusters, indicating immune evasion in the HR group.

This study has certain limitations. We only analyzed the BC data from TCGA for female patients. External GEO validation may have been influenced by imbalanced patient characteristics, necessitating further studies using additional datasets to confirm these findings.

In conclusion, the present study investigated the prognostic value of Pyro I mm lncRNAs in BC, leading to the development of a six-lncRNA prognostic risk model. By incorporating age, stage, and risk score, we constructed a nomogram model with strong predictive value for patient outcomes. The risk score calculated from ncRNA expression and risk coefficients demonstrated significant differences across different risk groups in terms of immune cell profiles, immune functionality, and immune checkpoint gene levels, suggesting distinct immune escape routes. These findings indicate the potential for improved HR patients with HR through immunotherapy. GSEA and GSVA revealed significant pathway and immune gene set differences between the risk groups, further supporting the unique biological characteristics of these groups. Validation using single-cell data revealed a scarcity of myeloid cells, T cells, B cells, and plasmablast clusters in the HR group, indicating immune evasion. Continued research is essential to uncover new therapeutic targets and guide personalized immunotherapy.

## Data Availability

The datasets presented in this study can be found in online repositories. The names of the repository/repositories and accession number(s) can be found in the article/**Supplementary Material**.
